# 5α-Dihydro­vespertilin acetate

**DOI:** 10.1107/S160053681000512X

**Published:** 2010-02-13

**Authors:** Michael Benn, Kanwal Nain Vohra, Masood Parvez

**Affiliations:** a Department of Chemistry, University of Calgary, 2500 University Drive NW, Calgary, Alberta, Canada T2N 1N4

## Abstract

In the title compound, C_24_H_36_O_4_ [systematic name: (20*S*)-3β-acet­oxy-16α-hydr­oxy-22,23-bis­nor-5α,17β-cholano(22-16)lac­tone], the three six-membered rings adopt classical chair conformations, while the five-membered rings are in envelope conformations. The ester group attached to ring *A* is in an equatorial position. Rings *A*/*B*, *B*/*C* and *C*/*D* are *trans*-fused, whereas rings *D*/*E* are *cis*-fused. The structure is devoid of any classical hydrogen bonds. However, non-classical inter- and intra­molecular hydrogen-bonding inter­actions of the type C—H⋯O are present in the structure.

## Related literature

For background to the synthesis, see: Vohra (1973 ▶). For spectroscopic data for 5α dihydro­vespertilin, see: Iglesias-Arteaga & Alvarado-Nuñes (2006 ▶). For a closely related structure, see: Novoa de Armas *et al.* (2000 ▶). For reference bond lengths, see: Allen *et al.* (1987 ▶). For puckering parameters, see: Cremer & Pople (1975 ▶).
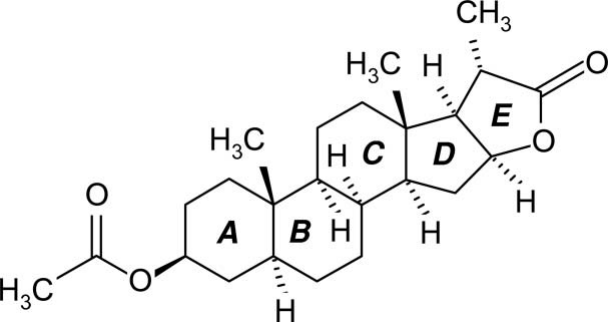


## Experimental

### 

#### Crystal data

C_24_H_36_O_4_
*M* *_r_* = 388.53
Orthorhombic, 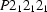

*a* = 6.4256 (3) Å
*b* = 9.6527 (6) Å
*c* = 34.953 (2) Å
*V* = 2167.9 (2) Å^3^
*Z* = 4
Mo *K*α radiation
μ = 0.08 mm^−1^
*T* = 173 K
0.30 × 0.08 × 0.02 mm

#### Data collection

Nonius Kappa geometry diffractometer with Bruker APEXII CCD
Absorption correction: multi-scan (*SORTAV*; Blessing, 1997 ▶) *T*_min_ = 0.977, *T*_max_ = 0.998
8349 measured reflections
2784 independent reflections
2554 reflections with *I* > 2σ(*I*)
*R*_int_ = 0.028

#### Refinement

*R*[*F* ^2^ > 2σ(*F* ^2^)] = 0.051
*wR*(*F* ^2^) = 0.119
*S* = 1.11
2784 reflections
256 parameters
H-atom parameters constrained
Δρ_max_ = 0.24 e Å^−3^
Δρ_min_ = −0.20 e Å^−3^

### 

Data collection: *COLLECT* (Nonius, 1998 ▶); cell refinement: *HKL* *DENZO* (Otwinowski & Minor, 1997 ▶); data reduction: *SCALEPACK* (Otwinowski & Minor, 1997 ▶); program(s) used to solve structure: *SHELXS97* (Sheldrick, 2008 ▶); program(s) used to refine structure: *SHELXL97* (Sheldrick, 2008 ▶); molecular graphics: *ORTEP-3 for Windows* (Farrugia, 1997 ▶); software used to prepare material for publication: *SHELXL97*.

## Supplementary Material

Crystal structure: contains datablocks global, I. DOI: 10.1107/S160053681000512X/lh2988sup1.cif

Structure factors: contains datablocks I. DOI: 10.1107/S160053681000512X/lh2988Isup2.hkl

Additional supplementary materials:  crystallographic information; 3D view; checkCIF report

## Figures and Tables

**Table 1:** Hydrogen-bond geometry (Å, °)

*D*—H⋯*A*	*D*—H	H⋯*A*	*D*⋯*A*	*D*—H⋯*A*
C16—H16⋯O1^i^	1.00	2.36	3.116 (3)	131
C18—H18*A*⋯O1	0.98	2.58	3.246 (3)	126

Symmetry code: (i) 
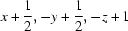
.

**Fig. 1.**
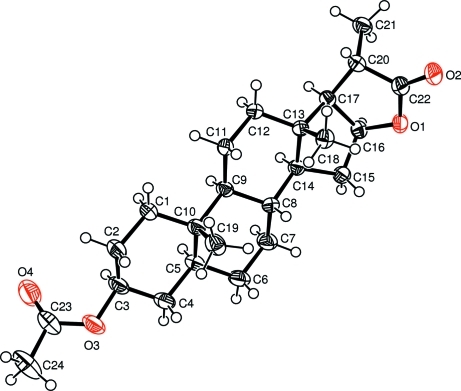
ORTEP-3 (Farrugia, 1997) drawing of the title compound with displacement ellipsoids plotted at 50% probability level.

## supplementary crystallographic information

### Comment

During some investigations of the thermolysis of the *N*-chloro and *N*-bromo derivatives of secondary amides in aqueous dioxane (1:4 v/v) it was discovered that a radical-chain reaction ensued in which the first-formed N-centred radical abstracted a hydrogen atom from a proximate site. In the case of intramolecular reactions a 6-membered transition state was strongly favoured, leading to a C-centred radical which abstracted a halogen from starting material in a chain-propagating step. Under the reaction conditions the intermediate C-halo product underwent intramolecular heterolysis with the carbonyl oxygen of the amide displacing the halogen and generating an iminolactone which in turn hydrolysed to produce a γ-lactone. This process afforded a method for the functionalisation of chemically unactivated sites within the steroid nucleus as illustrated by its application to the synthesis of 5α-dihydrovespertilin acetate (I) from the 3-*O*-acetate of *N*-chloro-*N*-methyl-5α-bisnorcholanamide (Vohra, 1973).

The molecular structure of (I) is presented in Fig. 1. The molecule contains three six-membered rings, A, B and C and two five-membered rings, D and E. The rings A/B, B/C and C/D are *trans*-fused whereas the rings D/E are *cis*-fused. The rings A–C adopt chair conformations. The puckering parameters (Cremer & Pople, 1975) for the rings A to C are: *Q* = 0.569 (3), 0.590 (3), 0.571 (3) Å, θ = 4.6 (3), 4.4 (3), 7.3 (2)° and φ = 305 (4), 272 (2), 248 (2)°, respectively. The rings D and E adopt envelope conformations with C13 and C17 0.697 (4) and 0.398 (4) Å, out of the mean-planes formed by the remaining ring atoms, respectively. The ester group attached to the ring A is in an equatorial position. The bond distances (Allen *et al.*, 1987) and angles in (I) are as expected. The structure is devoid of any classical hydrogen bonds. However, non-classical inter and intra molecular hydrogen bonding interactions of the type C—H···O invoving O1 are present in the structure (Table 1). The crystal structure of a compound very closely related to (I), 3β-acetoxy-5α,6β-dihydroxy-bisnorcholanic acid 22,16-lactone, has been reported (Novoa de Armas *et al.*, 2000).

### Experimental

A solution of *N*-chloro-*N*-methyl-5α-bisnorcholanamide acetate (500 mg, 1.14 mmol), prepared from the parent amide by treatment with excess t-butyl hypochlorite in the dark, in aqueous 1,4-dioxane (1:4 v/v) (50 ml) containing calcium carbonate (2.5 g) and dibenzoyl peroxide (20 mg) was heated to 358 K (bath) under an atmosphere of nitrogen. After 2 hr a test for active chlorine (starch-KI paper) was negative. The mixture was filtered and the filter cake washed with dioxane (2 × 25 ml). The combined filtrate and washings were evaporated to dryness (Rotovap) and the residue separated by PTLC on silica gel 60 F254 (Merck) (1 m × 20 cm × 2 mm) using EtOAc–PhH (1:1) v/v for development to give as the major product (20*S*)-3β-acetoxy-16α-hydroxy-22,23-bisnor-5α,17β-cholano(22-16)lactone (181 mg, 0.53 mmol, 46%), m.p. 492–493 K, with ^1^H and ^13^C-NMR as reported for this compound, 5α dihydrovespertilin (Iglesias-Arteaga & Alvarado-Nuñes, 2006). Suitable crystals of the title compound for X-ray study were grown from an aqueous solution of ethanol (ca 1:20) in the form of plates.

### Refinement

An absolute structure could not be established reliably because of insufficient anomalous scattering effects. Therefore, Friedel pairs (1738) were merged. The H atoms were included in the refinements at geometrically idealized positions with C—H distances = 0.98, 0.99 and 1.00 Å for methyl, methylene and methine type H atoms, respectively. The H atoms were assigned U_iso_ = 1.5 and 1.2 times U_eq_ of the methyl and non-methyl C atoms to which they were bonded. H atoms bonded to C24 were disordered over six sites with equal site occupancy factors. The final difference map was free of chemically significant features.

### Crystal data

**Table:**

C_24_H_36_O_4_	*D*_x_ = 1.190 Mg m^−^^3^
*M**_r_* = 388.53	Melting point = 492–493 K
Orthorhombic, *P*2_1_2_1_2_1_	Mo *K*α radiation, λ = 0.71073 Å
Hall symbol: P 2ac 2ab	Cell parameters from 2395 reflections
*a* = 6.4256 (3) Å	θ = 1.0–27.5°
*b* = 9.6527 (6) Å	µ = 0.08 mm^−^^1^
*c* = 34.953 (2) Å	*T* = 173 K
*V* = 2167.9 (2) Å^3^	Plate, colourless
*Z* = 4	0.30 × 0.08 × 0.02 mm
*F*(000) = 848	

### Data collection

**Table:**

Nonius APEXII CCD diffractometer	2784 independent reflections
Radiation source: fine-focus sealed tube	2554 reflections with *I* > 2σ(*I*)
graphite	*R*_int_ = 0.028
φ and ω scans	θ_max_ = 27.5°, θ_min_ = 2.7°
Absorption correction: multi-scan (*SORTAV*; Blessing, 1997)	*h* = −8→8
*T*_min_ = 0.977, *T*_max_ = 0.998	*k* = −12→12
8349 measured reflections	*l* = −44→45

### Refinement

**Table:**

Refinement on *F*^2^	Primary atom site location: structure-invariant direct methods
Least-squares matrix: full	Secondary atom site location: difference Fourier map
*R*[*F*^2^ > 2σ(*F*^2^)] = 0.051	Hydrogen site location: inferred from neighbouring sites
*wR*(*F*^2^) = 0.119	H-atom parameters constrained
*S* = 1.11	*w* = 1/[σ^2^(*F*_o_^2^) + (0.037*P*)^2^ + 1.01*P*] where *P* = (*F*_o_^2^ + 2*F*_c_^2^)/3
2784 reflections	(Δ/σ)_max_ < 0.001
256 parameters	Δρ_max_ = 0.24 e Å^−^^3^
0 restraints	Δρ_min_ = −0.20 e Å^−^^3^

### Special details

**Table:**

Geometry. All esds (except the esd in the dihedral angle between two l.s. planes) are estimated using the full covariance matrix. The cell esds are taken into account individually in the estimation of esds in distances, angles and torsion angles; correlations between esds in cell parameters are only used when they are defined by crystal symmetry. An approximate (isotropic) treatment of cell esds is used for estimating esds involving l.s. planes.
Refinement. Refinement of *F*^2^ against ALL reflections. The weighted *R*-factor wR and goodness of fit *S* are based on *F*^2^, conventional *R*-factors *R* are based on *F*, with *F* set to zero for negative *F*^2^. The threshold expression of *F*^2^ > σ(*F*^2^) is used only for calculating *R*-factors(gt) etc. and is not relevant to the choice of reflections for refinement. *R*-factors based on *F*^2^ are statistically about twice as large as those based on *F*, and *R*- factors based on ALL data will be even larger.An absolute structure using Flack method [Flack H D (1983), Acta Cryst. A39, 876-881] could not be established reliably becuase of insufficient anomalous scattering effects. Therefore, Friedel pairs (1738) were merged.

### Fractional atomic coordinates and isotropic or equivalent isotropic displacement parameters (Å^2^)

**Table:**

	*x*	*y*	*z*	*U*_iso_*/*U*_eq_	Occ. (<1)
O1	−0.0207 (3)	0.09610 (19)	0.50200 (5)	0.0334 (4)	
O2	−0.1447 (3)	−0.0949 (2)	0.47447 (6)	0.0434 (5)	
O3	0.8680 (4)	0.1358 (3)	0.78266 (5)	0.0487 (6)	
O4	1.2141 (4)	0.0994 (3)	0.78197 (7)	0.0667 (8)	
C1	0.8182 (4)	−0.0137 (3)	0.68247 (7)	0.0358 (6)	
H1A	0.9327	0.0336	0.6687	0.043*	
H1B	0.8152	−0.1113	0.6738	0.043*	
C2	0.8633 (5)	−0.0097 (3)	0.72580 (7)	0.0407 (7)	
H2A	0.7571	−0.0650	0.7395	0.049*	
H2B	1.0012	−0.0515	0.7309	0.049*	
C3	0.8604 (5)	0.1379 (3)	0.74049 (7)	0.0402 (7)	
H3	0.9840	0.1890	0.7304	0.048*	
C4	0.6643 (5)	0.2149 (3)	0.73005 (7)	0.0418 (7)	
H4A	0.6782	0.3132	0.7378	0.050*	
H4B	0.5453	0.1747	0.7442	0.050*	
C5	0.6209 (5)	0.2071 (3)	0.68664 (7)	0.0349 (6)	
H5	0.7418	0.2519	0.6735	0.042*	
C6	0.4282 (5)	0.2905 (3)	0.67576 (8)	0.0471 (8)	
H6A	0.4447	0.3872	0.6847	0.057*	
H6B	0.3047	0.2506	0.6886	0.057*	
C7	0.3937 (5)	0.2900 (3)	0.63221 (8)	0.0435 (7)	
H7A	0.2613	0.3379	0.6263	0.052*	
H7B	0.5079	0.3417	0.6196	0.052*	
C8	0.3860 (4)	0.1422 (3)	0.61610 (6)	0.0295 (5)	
H8	0.2630	0.0937	0.6274	0.035*	
C9	0.5849 (4)	0.0615 (3)	0.62748 (6)	0.0274 (5)	
H9	0.7049	0.1168	0.6176	0.033*	
C10	0.6112 (4)	0.0558 (3)	0.67195 (6)	0.0294 (5)	
C11	0.5995 (4)	−0.0820 (3)	0.60811 (7)	0.0308 (5)	
H11A	0.7385	−0.1222	0.6133	0.037*	
H11B	0.4941	−0.1443	0.6196	0.037*	
C12	0.5651 (4)	−0.0757 (3)	0.56452 (7)	0.0297 (5)	
H12A	0.6806	−0.0237	0.5525	0.036*	
H12B	0.5651	−0.1708	0.5539	0.036*	
C13	0.3596 (4)	−0.0054 (2)	0.55515 (6)	0.0256 (5)	
C14	0.3631 (4)	0.1415 (2)	0.57245 (6)	0.0270 (5)	
H14	0.4904	0.1877	0.5621	0.032*	
C15	0.1770 (4)	0.2139 (3)	0.55379 (7)	0.0332 (6)	
H15A	0.1951	0.3158	0.5538	0.040*	
H15B	0.0456	0.1904	0.5670	0.040*	
C16	0.1799 (4)	0.1556 (3)	0.51274 (7)	0.0296 (5)	
H16	0.2231	0.2286	0.4941	0.035*	
C17	0.3339 (4)	0.0331 (2)	0.51221 (6)	0.0267 (5)	
H17	0.4706	0.0640	0.5015	0.032*	
C18	0.1768 (4)	−0.0934 (3)	0.56960 (7)	0.0316 (5)	
H18A	0.0453	−0.0482	0.5628	0.047*	
H18B	0.1857	−0.1028	0.5975	0.047*	
H18C	0.1825	−0.1854	0.5578	0.047*	
C19	0.4331 (5)	−0.0268 (3)	0.69033 (8)	0.0404 (7)	
H19A	0.3001	0.0032	0.6794	0.061*	
H19B	0.4324	−0.0106	0.7180	0.061*	
H19C	0.4532	−0.1258	0.6853	0.061*	
C20	0.2312 (4)	−0.0705 (3)	0.48481 (7)	0.0286 (5)	
H20	0.2516	−0.1676	0.4941	0.034*	
C21	0.3096 (4)	−0.0551 (3)	0.44337 (7)	0.0375 (6)	
H21A	0.2275	−0.1150	0.4265	0.056*	
H21B	0.4564	−0.0820	0.4421	0.056*	
H21C	0.2945	0.0416	0.4352	0.056*	
C22	0.0030 (4)	−0.0311 (3)	0.48607 (7)	0.0321 (6)	
C23	1.0529 (6)	0.1106 (4)	0.79906 (8)	0.0500 (8)	
C24	1.0304 (7)	0.0994 (4)	0.84196 (8)	0.0692 (12)	
H24A	0.8840	0.1120	0.8490	0.104*	0.50
H24B	1.1149	0.1712	0.8543	0.104*	0.50
H24C	1.0777	0.0079	0.8504	0.104*	0.50
H24D	1.1670	0.0820	0.8535	0.104*	0.50
H24E	0.9362	0.0228	0.8482	0.104*	0.50
H24F	0.9733	0.1861	0.8521	0.104*	0.50

### Atomic displacement parameters (Å^2^)

**Table:**

	*U*^11^	*U*^22^	*U*^33^	*U*^12^	*U*^13^	*U*^23^
O1	0.0272 (9)	0.0343 (9)	0.0388 (9)	0.0019 (9)	−0.0071 (8)	−0.0022 (8)
O2	0.0321 (10)	0.0468 (11)	0.0514 (11)	−0.0070 (10)	−0.0072 (9)	−0.0082 (10)
O3	0.0518 (12)	0.0723 (15)	0.0220 (8)	−0.0066 (13)	−0.0015 (9)	−0.0056 (9)
O4	0.0518 (15)	0.101 (2)	0.0472 (13)	−0.0075 (17)	−0.0111 (11)	−0.0020 (14)
C1	0.0372 (14)	0.0467 (16)	0.0236 (11)	0.0070 (14)	−0.0036 (11)	−0.0019 (11)
C2	0.0420 (15)	0.0546 (17)	0.0256 (11)	0.0005 (16)	−0.0057 (12)	−0.0018 (12)
C3	0.0413 (15)	0.0593 (18)	0.0198 (11)	−0.0058 (16)	0.0006 (11)	−0.0037 (11)
C4	0.0459 (16)	0.0522 (16)	0.0272 (12)	−0.0016 (16)	0.0009 (12)	−0.0116 (12)
C5	0.0413 (15)	0.0400 (14)	0.0234 (11)	0.0000 (14)	0.0007 (11)	−0.0054 (10)
C6	0.060 (2)	0.0486 (17)	0.0327 (13)	0.0151 (17)	−0.0029 (14)	−0.0143 (13)
C7	0.0559 (18)	0.0389 (15)	0.0357 (13)	0.0149 (15)	−0.0072 (14)	−0.0090 (12)
C8	0.0292 (12)	0.0350 (13)	0.0245 (10)	0.0043 (12)	0.0032 (10)	−0.0034 (10)
C9	0.0298 (12)	0.0319 (12)	0.0206 (10)	−0.0013 (11)	0.0018 (9)	−0.0005 (9)
C10	0.0307 (13)	0.0358 (13)	0.0216 (10)	−0.0014 (12)	0.0013 (10)	−0.0013 (10)
C11	0.0317 (13)	0.0333 (13)	0.0274 (11)	0.0096 (12)	−0.0047 (10)	−0.0030 (10)
C12	0.0292 (12)	0.0357 (13)	0.0241 (11)	0.0052 (11)	−0.0002 (9)	−0.0040 (10)
C13	0.0250 (11)	0.0285 (11)	0.0232 (10)	0.0002 (10)	0.0019 (9)	−0.0003 (9)
C14	0.0291 (12)	0.0279 (11)	0.0239 (10)	0.0014 (11)	0.0008 (10)	−0.0006 (9)
C15	0.0373 (14)	0.0303 (12)	0.0321 (12)	0.0074 (12)	−0.0040 (11)	−0.0021 (11)
C16	0.0301 (13)	0.0292 (12)	0.0294 (11)	−0.0004 (11)	−0.0035 (10)	0.0016 (10)
C17	0.0264 (12)	0.0297 (11)	0.0239 (10)	−0.0033 (11)	0.0009 (9)	0.0007 (10)
C18	0.0287 (12)	0.0335 (13)	0.0324 (12)	−0.0024 (12)	0.0021 (10)	0.0043 (11)
C19	0.0386 (15)	0.0549 (18)	0.0278 (12)	−0.0098 (15)	0.0005 (11)	0.0057 (13)
C20	0.0323 (13)	0.0299 (12)	0.0238 (10)	−0.0023 (11)	−0.0012 (10)	−0.0013 (10)
C21	0.0382 (14)	0.0466 (15)	0.0277 (11)	−0.0043 (14)	0.0017 (11)	−0.0054 (11)
C22	0.0319 (13)	0.0337 (13)	0.0306 (12)	−0.0022 (12)	−0.0008 (11)	0.0016 (11)
C23	0.063 (2)	0.0545 (19)	0.0323 (14)	−0.0132 (19)	−0.0128 (15)	−0.0006 (14)
C24	0.093 (3)	0.083 (3)	0.0313 (15)	−0.030 (3)	−0.0172 (17)	0.0083 (17)

### Geometric parameters (Å, °)

**Table:**

O1—C22	1.357 (3)	C11—H11B	0.9900
O1—C16	1.460 (3)	C12—C13	1.520 (3)
O2—C22	1.202 (3)	C12—H12A	0.9900
O3—C23	1.342 (4)	C12—H12B	0.9900
O3—C3	1.475 (3)	C13—C18	1.535 (3)
O4—C23	1.200 (4)	C13—C14	1.542 (3)
C1—C10	1.535 (4)	C13—C17	1.555 (3)
C1—C2	1.542 (3)	C14—C15	1.531 (3)
C1—H1A	0.9900	C14—H14	1.0000
C1—H1B	0.9900	C15—C16	1.541 (3)
C2—C3	1.514 (4)	C15—H15A	0.9900
C2—H2A	0.9900	C15—H15B	0.9900
C2—H2B	0.9900	C16—C17	1.543 (4)
C3—C4	1.508 (4)	C16—H16	1.0000
C3—H3	1.0000	C17—C20	1.534 (3)
C4—C5	1.545 (3)	C17—H17	1.0000
C4—H4A	0.9900	C18—H18A	0.9800
C4—H4B	0.9900	C18—H18B	0.9800
C5—C6	1.525 (4)	C18—H18C	0.9800
C5—C10	1.549 (4)	C19—H19A	0.9800
C5—H5	1.0000	C19—H19B	0.9800
C6—C7	1.539 (4)	C19—H19C	0.9800
C6—H6A	0.9900	C20—C22	1.515 (4)
C6—H6B	0.9900	C20—C21	1.541 (3)
C7—C8	1.534 (4)	C20—H20	1.0000
C7—H7A	0.9900	C21—H21A	0.9800
C7—H7B	0.9900	C21—H21B	0.9800
C8—C14	1.533 (3)	C21—H21C	0.9800
C8—C9	1.548 (3)	C23—C24	1.510 (4)
C8—H8	1.0000	C24—H24A	0.9800
C9—C11	1.545 (3)	C24—H24B	0.9800
C9—C10	1.565 (3)	C24—H24C	0.9800
C9—H9	1.0000	C24—H24D	0.9800
C10—C19	1.536 (4)	C24—H24E	0.9800
C11—C12	1.541 (3)	C24—H24F	0.9800
C11—H11A	0.9900		
			
C22—O1—C16	111.24 (19)	C18—C13—C17	111.6 (2)
C23—O3—C3	117.3 (2)	C14—C13—C17	99.24 (18)
C10—C1—C2	112.8 (2)	C15—C14—C8	119.8 (2)
C10—C1—H1A	109.0	C15—C14—C13	103.99 (19)
C2—C1—H1A	109.0	C8—C14—C13	113.3 (2)
C10—C1—H1B	109.0	C15—C14—H14	106.3
C2—C1—H1B	109.0	C8—C14—H14	106.3
H1A—C1—H1B	107.8	C13—C14—H14	106.3
C3—C2—C1	110.7 (2)	C14—C15—C16	102.75 (19)
C3—C2—H2A	109.5	C14—C15—H15A	111.2
C1—C2—H2A	109.5	C16—C15—H15A	111.2
C3—C2—H2B	109.5	C14—C15—H15B	111.2
C1—C2—H2B	109.5	C16—C15—H15B	111.2
H2A—C2—H2B	108.1	H15A—C15—H15B	109.1
O3—C3—C4	106.0 (2)	O1—C16—C15	111.9 (2)
O3—C3—C2	109.0 (2)	O1—C16—C17	105.15 (19)
C4—C3—C2	113.1 (3)	C15—C16—C17	107.39 (19)
O3—C3—H3	109.5	O1—C16—H16	110.7
C4—C3—H3	109.5	C15—C16—H16	110.7
C2—C3—H3	109.5	C17—C16—H16	110.7
C3—C4—C5	111.4 (2)	C20—C17—C16	103.4 (2)
C3—C4—H4A	109.4	C20—C17—C13	119.5 (2)
C5—C4—H4A	109.4	C16—C17—C13	103.87 (18)
C3—C4—H4B	109.4	C20—C17—H17	109.8
C5—C4—H4B	109.4	C16—C17—H17	109.8
H4A—C4—H4B	108.0	C13—C17—H17	109.8
C6—C5—C4	111.4 (2)	C13—C18—H18A	109.5
C6—C5—C10	112.5 (2)	C13—C18—H18B	109.5
C4—C5—C10	112.3 (2)	H18A—C18—H18B	109.5
C6—C5—H5	106.7	C13—C18—H18C	109.5
C4—C5—H5	106.7	H18A—C18—H18C	109.5
C10—C5—H5	106.7	H18B—C18—H18C	109.5
C5—C6—C7	111.2 (2)	C10—C19—H19A	109.5
C5—C6—H6A	109.4	C10—C19—H19B	109.5
C7—C6—H6A	109.4	H19A—C19—H19B	109.5
C5—C6—H6B	109.4	C10—C19—H19C	109.5
C7—C6—H6B	109.4	H19A—C19—H19C	109.5
H6A—C6—H6B	108.0	H19B—C19—H19C	109.5
C8—C7—C6	111.8 (2)	C22—C20—C17	103.6 (2)
C8—C7—H7A	109.3	C22—C20—C21	108.6 (2)
C6—C7—H7A	109.3	C17—C20—C21	112.5 (2)
C8—C7—H7B	109.3	C22—C20—H20	110.6
C6—C7—H7B	109.3	C17—C20—H20	110.6
H7A—C7—H7B	107.9	C21—C20—H20	110.6
C14—C8—C7	111.9 (2)	C20—C21—H21A	109.5
C14—C8—C9	109.43 (19)	C20—C21—H21B	109.5
C7—C8—C9	110.3 (2)	H21A—C21—H21B	109.5
C14—C8—H8	108.4	C20—C21—H21C	109.5
C7—C8—H8	108.4	H21A—C21—H21C	109.5
C9—C8—H8	108.4	H21B—C21—H21C	109.5
C11—C9—C8	112.9 (2)	O2—C22—O1	120.9 (2)
C11—C9—C10	113.4 (2)	O2—C22—C20	128.7 (2)
C8—C9—C10	111.22 (19)	O1—C22—C20	110.4 (2)
C11—C9—H9	106.2	O4—C23—O3	124.6 (3)
C8—C9—H9	106.2	O4—C23—C24	124.8 (3)
C10—C9—H9	106.2	O3—C23—C24	110.6 (3)
C1—C10—C19	108.6 (2)	C23—C24—H24A	109.5
C1—C10—C5	107.3 (2)	C23—C24—H24B	109.5
C19—C10—C5	112.4 (2)	H24A—C24—H24B	109.5
C1—C10—C9	110.3 (2)	C23—C24—H24C	109.5
C19—C10—C9	110.7 (2)	H24A—C24—H24C	109.5
C5—C10—C9	107.5 (2)	H24B—C24—H24C	109.5
C12—C11—C9	112.9 (2)	C23—C24—H24D	109.5
C12—C11—H11A	109.0	H24A—C24—H24D	141.1
C9—C11—H11A	109.0	H24B—C24—H24D	56.3
C12—C11—H11B	109.0	H24C—C24—H24D	56.3
C9—C11—H11B	109.0	C23—C24—H24E	109.5
H11A—C11—H11B	107.8	H24A—C24—H24E	56.3
C13—C12—C11	110.8 (2)	H24B—C24—H24E	141.1
C13—C12—H12A	109.5	H24C—C24—H24E	56.3
C11—C12—H12A	109.5	H24D—C24—H24E	109.5
C13—C12—H12B	109.5	C23—C24—H24F	109.5
C11—C12—H12B	109.5	H24A—C24—H24F	56.3
H12A—C12—H12B	108.1	H24B—C24—H24F	56.3
C12—C13—C18	110.31 (19)	H24C—C24—H24F	141.1
C12—C13—C14	108.3 (2)	H24D—C24—H24F	109.5
C18—C13—C14	113.0 (2)	H24E—C24—H24F	109.5
C12—C13—C17	114.01 (19)		
			
C10—C1—C2—C3	−56.4 (3)	C7—C8—C14—C15	−57.9 (3)
C23—O3—C3—C4	−160.7 (3)	C9—C8—C14—C15	179.6 (2)
C23—O3—C3—C2	77.2 (4)	C7—C8—C14—C13	178.7 (2)
C1—C2—C3—O3	170.4 (2)	C9—C8—C14—C13	56.2 (3)
C1—C2—C3—C4	52.7 (3)	C12—C13—C14—C15	167.09 (19)
O3—C3—C4—C5	−172.0 (2)	C18—C13—C14—C15	−70.4 (2)
C2—C3—C4—C5	−52.6 (3)	C17—C13—C14—C15	47.9 (2)
C3—C4—C5—C6	−177.4 (3)	C12—C13—C14—C8	−61.2 (3)
C3—C4—C5—C10	55.4 (3)	C18—C13—C14—C8	61.3 (3)
C4—C5—C6—C7	176.4 (3)	C17—C13—C14—C8	179.6 (2)
C10—C5—C6—C7	−56.5 (3)	C8—C14—C15—C16	−165.0 (2)
C5—C6—C7—C8	54.1 (4)	C13—C14—C15—C16	−37.2 (2)
C6—C7—C8—C14	−177.0 (2)	C22—O1—C16—C15	−132.0 (2)
C6—C7—C8—C9	−55.0 (3)	C22—O1—C16—C17	−15.7 (2)
C14—C8—C9—C11	−49.6 (3)	C14—C15—C16—O1	126.4 (2)
C7—C8—C9—C11	−173.0 (2)	C14—C15—C16—C17	11.5 (3)
C14—C8—C9—C10	−178.4 (2)	O1—C16—C17—C20	24.1 (2)
C7—C8—C9—C10	58.2 (3)	C15—C16—C17—C20	143.4 (2)
C2—C1—C10—C19	−64.1 (3)	O1—C16—C17—C13	−101.4 (2)
C2—C1—C10—C5	57.6 (3)	C15—C16—C17—C13	17.9 (3)
C2—C1—C10—C9	174.4 (2)	C12—C13—C17—C20	91.2 (3)
C6—C5—C10—C1	176.5 (2)	C18—C13—C17—C20	−34.6 (3)
C4—C5—C10—C1	−56.8 (3)	C14—C13—C17—C20	−153.9 (2)
C6—C5—C10—C19	−64.2 (3)	C12—C13—C17—C16	−154.3 (2)
C4—C5—C10—C19	62.5 (3)	C18—C13—C17—C16	79.8 (2)
C6—C5—C10—C9	57.8 (3)	C14—C13—C17—C16	−39.5 (2)
C4—C5—C10—C9	−175.5 (2)	C16—C17—C20—C22	−23.4 (2)
C11—C9—C10—C1	56.3 (3)	C13—C17—C20—C22	91.3 (3)
C8—C9—C10—C1	−175.2 (2)	C16—C17—C20—C21	93.7 (2)
C11—C9—C10—C19	−63.9 (3)	C13—C17—C20—C21	−151.6 (2)
C8—C9—C10—C19	64.6 (3)	C16—O1—C22—O2	−178.0 (2)
C11—C9—C10—C5	173.0 (2)	C16—O1—C22—C20	0.3 (3)
C8—C9—C10—C5	−58.5 (3)	C17—C20—C22—O2	−166.6 (3)
C8—C9—C11—C12	50.2 (3)	C21—C20—C22—O2	73.6 (4)
C10—C9—C11—C12	177.8 (2)	C17—C20—C22—O1	15.2 (3)
C9—C11—C12—C13	−54.8 (3)	C21—C20—C22—O1	−104.6 (2)
C11—C12—C13—C18	−65.7 (3)	C3—O3—C23—O4	5.5 (5)
C11—C12—C13—C14	58.4 (3)	C3—O3—C23—C24	−175.0 (3)
C11—C12—C13—C17	167.8 (2)		

### Hydrogen-bond geometry (Å, °)

**Table:**

*D*—H···*A*	*D*—H	H···*A*	*D*···*A*	*D*—H···*A*
C16—H16···O1^i^	1.00	2.36	3.116 (3)	131
C18—H18A···O1	0.98	2.58	3.246 (3)	126

Symmetry codes: (i) *x*+1/2, −*y*+1/2, −*z*+1.
